# Pediatric Low-Grade Gliomas

**DOI:** 10.3390/cancers12051152

**Published:** 2020-05-04

**Authors:** Kelly L. Collins, Ian F. Pollack

**Affiliations:** Department of Pediatric Neurosurgery, University of Pittsburgh, Pittsburgh, PA 15224, USA; pollaci@upmc.edu

**Keywords:** pediatric low-grade glioma, pilocytic astrocytoma, diffuse astrocytoma, pleomorphic xanthoastrocytoma, subependymal giant cell astrocytoma, neuroepithelial tumor, BRAF mutation, molecularly-targeted therapy

## Abstract

Brain tumors constitute the largest source of oncologic mortality in children and low-grade gliomas are among most common pediatric central nervous system tumors. Pediatric low-grade gliomas differ from their counterparts in the adult population in their histopathology, genetics, and standard of care. Over the past decade, an increasingly detailed understanding of the molecular and genetic characteristics of pediatric brain tumors led to tailored therapy directed by integrated phenotypic and genotypic parameters and the availability of an increasing array of molecular-directed therapies. Advances in neuroimaging, conformal radiation therapy, and conventional chemotherapy further improved treatment outcomes. This article reviews the current classification of pediatric low-grade gliomas, their histopathologic and radiographic features, state-of-the-art surgical and adjuvant therapies, and emerging therapies currently under study in clinical trials.

## 1. Introduction

Brain tumors are the most common solid tumors in children and are the leading cause of childhood cancer-related mortality [1]. The distribution, pathology, molecular characteristics, and treatment strategies for pediatric brain tumors have important differences in comparison to those of the adult population. Similar to adults, gliomas comprise the majority of supratentorial intraparenchymal tumors in children [1]. However, while most intraparenchymal lesions in adults are malignant gliomas, low-grade gliomas predominate in children. Where adult low-grade gliomas routinely evolve into higher-grade lesions [2,3,4,5], malignant transformation occurs less frequently in childhood gliomas, a consequence of fundamental molecular and genetic differences between these groups of tumors. For example, adult low-grade astrocytomas and the higher grade gliomas which arise from malignant progression often possess *IDH1* or *IDH2* gene mutations [4,5,6,7]. *ATRX*, 1p19q codeletion, whereas *IDH* mutations are rare in childhood low-grade gliomas outside of adolescence [7,8]. Similarly, adult malignant gliomas frequently exhibit mutations in *TP53*, which is seen much less frequently in pediatric low-grade gliomas.

Due to the increasingly detailed understanding of the molecular and genetic characteristics of pediatric brain tumors, therapy is directed by tumor categorization based on integrated phenotypic and genotypic parameters, and an increasing array of molecular-directed therapies are available. The World Health Organization (WHO) now recognizes many subsets of tumors which respond to distinct therapies [9]. For example, the treatment of pilocytic astrocytomas dramatically changed when it was determined that the vast majority result from genetic alterations in the mitogen-activated protein kinase (MAPK) signaling pathway, most often in the *BRAF* gene, which serves as a therapeutic target [10,11,12,13,14,15]. An improved understanding of the molecular and genetic profile of many other tumors led to multiple open avenues of investigation for targeted therapies.

Surgical resection remains the mainstay of treatment for low-grade gliomas. Since 1970, the five-year relative survival rate for children with central nervous system (CNS )tumors improved from 57% to 74% [16]. As survival rates for children after the resection of favorable-risk tumors in particular improved, the clinical standard of post-resection radiation and conventional chemotherapy was replaced with a more nuanced approach to reduce the morbidity of adjuvant therapy. Risk-adapted treatment protocols were adopted for prognostically favorable tumors and molecularly targeted therapies broadened the medical options for both poor- and favorable-risk lesions. This article reviews the current classification of pediatric low-grade gliomas, state-of-the-art surgical and adjuvant therapies, and emerging therapies currently under study in clinical trials.

## 2. Histopathology and Molecular Pathogenesis

Low-grade gliomas are defined as WHO Grade I and II tumors with at least some component of glial cell lineage. These are separated histopathologically into several groups based on the “integrated” phenotypic and genotypic parameters of the 2016 World Health Organization classification of tumors of the central nervous system [9]. These groups are reviewed in Table 1.

For a comprehensive review of all subtypes of low-grade glioma and histopathological images, the reader is referred to the 2016 World Health Organization classification of tumors of the central nervous system [9]. A select subset of low-grade gliomas which arise frequently in the pediatric population is reviewed below.

### 2.1. Diffuse Astrocytic Tumors

**Figure 1:**
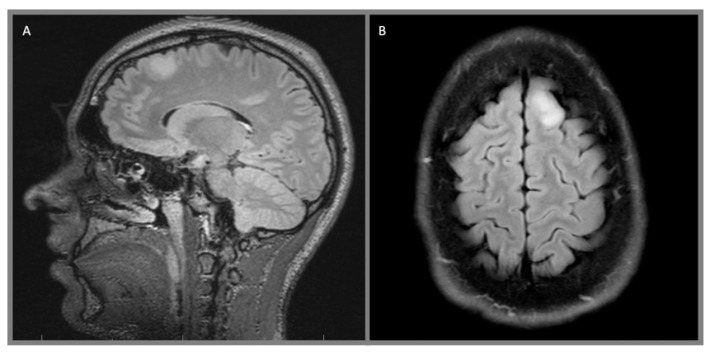
Left frontal diffuse astrocytoma, WHO 2: (**A**) sagittal T2 Fluid-attenuated inversion recovery (FLAIR) and (**B**) axial T2 FLAIR sequences demonstrate a diffusely infiltrating, hyperintense lesion in the left superior frontal gyrus.

Diffuse astrocytomas are WHO Grade II lesions characterized by the diffuse infiltration of normal brain parenchyma by well-differentiated neoplastic astrocytes (Figure 1). Several variants exist, including fibrillary astrocytomas which contain cells with enlongated, atypical nuclei, scant cytoplasm, and glial fibrillary acidic protein (GFAP)-positive fibrillary process, as well as the gemistocytic astrocytoma, which is composed of large cells with eccentric nuclei, GFAP-positive stocky processes, and eosinophilic cytoplasm. Mitotic activity is absent in both subtypes [17]. Amplification and/or rearrangement of *MYB/MYBL1* may be identified [18,19,20].

### 2.2. Other Astrocytic Tumors

**Figure 2:**
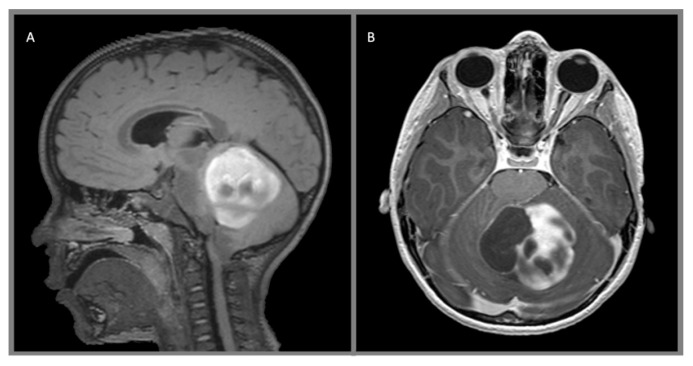
Juvenile pilocytic astrocytoma, WHO 1: (**A**) sagittal T2 FLAIR with contrast and (**B**) axial T1 with contrast demonstrate a heterogeneous, multicystic, avidly enhancing mass arising from the left cerebellar hemisphere.

Pilocytic astrocytomas are WHO Grade I lesions characterized by regions of compact bipolar astrocytes with long GFAP-positive processes alternating with more cellularly sparse cystic areas within well-circumscribed borders (Figure 2). Eosinophilic granular bodies, Rosenthal fibers, and microcysts are commonly seen. Mitotic figures, leptomeningeal infiltration, and glomeroid vascular proliferation are also frequently observed and do not increase the grade of the tumor [21]. These are slow-growing tumors with rare malignant transformation and are often located in midline structures rather than the cerebral or cerebellar hemispheres [21].

Various alterations in the *BRAF* gene or other regulators of MAPK signaling are common, including activating mutations such as *BRAF*^V600E^, translocations such as that between the *BRAF* gene and the *KIAA1549* gene that produce a constitutively active variant, or the neurofibromin mutation seen in neurofibromatosis-1 (NF-1) related tumors, all of which affect the MAPK signaling pathway [10,11,12,13,14,15]. Altogether, more than 80% of pilocytic astrocytomas have alterations in at least one component of the MAPK signaling pathway, providing multiple possible novel molecular targets for therapeutic intervention and underscoring the potential benefit provided by the molecular characterization of these tumors.

Pleomorphic xanthoastrocytomas carry a WHO Grade II classification. These lesions are often cystic and are characterized histologically by dense cellularity and nuclear atypia with pleomorphism and multinucleation, which can lead to misdiagnosis as a higher-grade lesion. However, these tumors should have a low mitotic index. Abundant lipid-rich “xanthomatous” astrocytes, extracellular reticulin, eosinophilic granular bodies, and lymphocytic infiltrate are often seen. Most often, these tumors involve the superficial temporal or parietal cortices in teenagers and young adults, with leptomeningeal invasion being common. *BRAF*^V600E^ mutations and 9p21 (*CDKN2A/B*) deletions may be seen [9].

Subependymal giant cell astrocytomas are WHO Grade I tumors that arise near the foramen of Monro and are strongly associated with tuberous sclerosis. These lesions were historically identified when they grew large enough to produce symptomatic obstructive hydrocephalus. However, surveillance imaging of patients with diagnosed tuberous sclerosis now identifies many of these lesions at an earlier stage. Histologically, these tumors contain large gemistocytic, spindled, and ganglion-cell like astrocytes, however, immunoreactivity for both glial and neuronal markers is often observed. Perivascular pseudopalisading may be seen, but other higher-grade features such as mitoses are not. Dysregulation of mTOR signaling was found to underlie the development of subependymal giant cell astrocytomas in tuberous sclerosis, which provided a basis for logical strategies for mTOR-directed molecular-targeted therapy [22,23].

### 2.3. Neuronal and Mixed Neuronal–Glial Tumors

**Figure 3:**
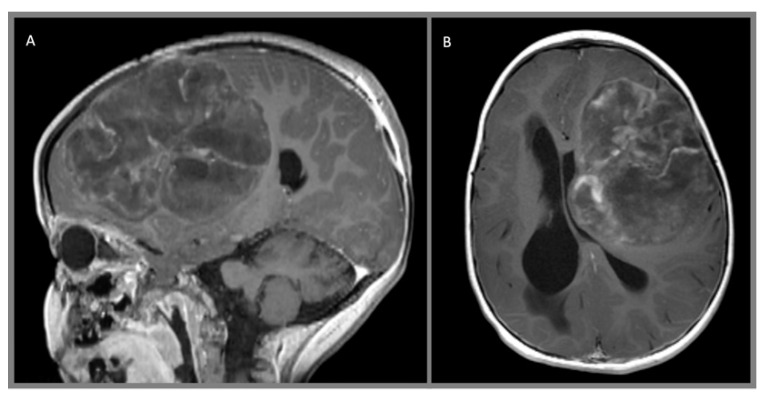
Giant ganglioglioma with extensive chondroid metaplasia, WHO I, arising from the left frontal lobe demonstrates multinodular architecture and sparse, heterogeneous contrast enhancement on these (**A**) sagittal and (**B**) axial T1 MPRAGE contrast-enhanced images.

Each of the benign neuroepithelial tumors has distinct morphological characteristics. Gangliogliomas are WHO Grade I lesions composed of highly differentiated binucleated ganglion cells in a background of astrocytes or occasionally oligodendrocytes (Figure 3) [24]. While both cell types are aberrant and considered neoplastic, it is the gliomatous component of the tumor that is responsible for the cell proliferation, growth, and clinical outcome of the tumor [21]. Alterations of *BRAF*, particularly *BRAF*^V600E^, or other regulators of the MAPK pathway were noted [25,26]. A small subset exhibit *CDKN2A* deletion [26].

**Figure 4:**
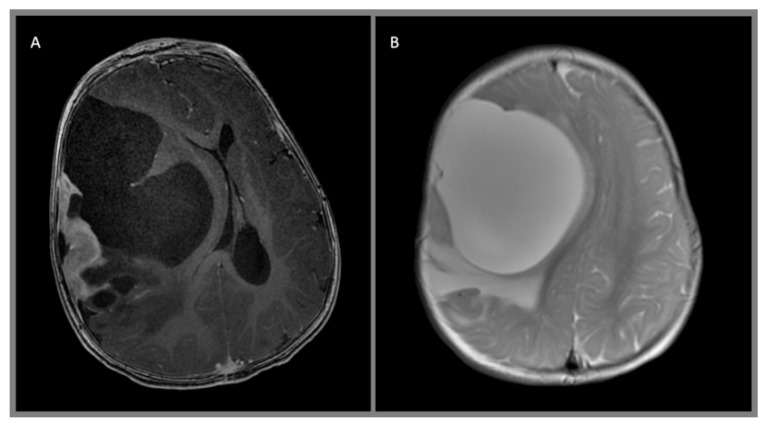
Desmoplastic infantile ganglioglioma, WHO I: (**A**) axial T1 MPRAGE contrast enhanced and (**B**) axial T2 images demonstrate a large right frontoparietal mass with a large cystic component and a superficial enhancing nodule with adjacent perilesional parenchymal edema.

Desmoplastic infantile gangliogliomas (DIG) are WHO Grade I mixed glial–neuronal neoplasms which present as large lesions in infants (Figure 4). They are usually cortically located and are often cystic. Histologically, these tumors exhibit a dense, fibrous, desmoplastic stroma containing a mixture of neuroepithelial cells with both astrocytic and neuronal differentiation [21,27]. These tumors frequently have *BRAF*^V600E^ mutations [28].

**Figure 5:**
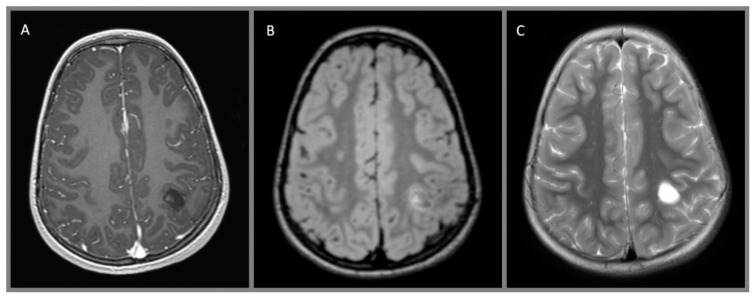
Dysembryoplastic neuroepithelial tumor, WHO I: (**A**) axial T1 MPRAGE with contrast, (**B**) axial T2 FLAIR with contrast, and (**C**) axial T2 without contrast demonstrate a small cystic lesion abutting the central sulcus in the left hemisphere. There is a small nodule and rim of enhancement at the medial aspect of the tumor.

Dysembryoplastic neuroepithelial tumors (DNET) are WHO Grade I lesions which arise in children and young adults, often in the temporal lobe, and commonly present with seizures and/or medically refractory epilepsy (Figure 5). The tumors are frequently found adjacent to regions of cortical dysplasia [21]. Histopathologically, DNETs are composed of nodules of oligodendroglial-like cells and/or focal cortical dysplasia intermixed with a looser textured component containing “floating neurons” in a mucinous matrix [21]. The glial nodules are generally GFAP-positive and the neuronal cell processes show synaptophysin positivity. *FGFR1* alterations and MAPK pathway activation are frequent [29]. 

## 3. Clinical Presentation

Low-grade gliomas typically present with seizures or focal neurologic deficits, such as weakness, sensory loss, language difficulty, visual impairment, cognitive difficulty, personality change, or a change in academic or athletic performance [30]. Due to the indolent nature of low-grade lesions, the symptoms are often insidious and slowly progress over the course of months or years. These tumors may also be discovered incidentally on neuroimaging acquired for another reason. Benign neuroepithelial tumors characteristically present with medically refractory focal seizures. Signs of increased intracranial pressure usually only occur when lesions obstruct ventricular outflow and result in hydrocephalus, or in rare cases when intratumoral hemorrhage results in a sudden increase in tumoral volume. For example, subependymal giant cell astrocytomas classically cause obstructive hydrocephalus by obstructing ventricular outflow at the foramen of Monro. In a notable exception, desmoplastic infantile gangliogliomas present as large masses in infancy with failure to thrive and signs of increased intracranial pressure, such as macrocephaly, full fontanelle, and the “setting sun” phenomenon. Optic gliomas manifest differently based on tumor location and age of the child at presentation. Tumors involving a single optic nerve can present with proptosis and unilateral vision loss. Tumors of the chiasm present with bilateral visual deterioration, esotropia, nystagmus, poor visual fixation, or optic atrophy on fundoscopy. Tumors which invade both the chiasm and hypothalamus may additionally present with signs of increased intracranial pressure (ICP), endocrine abnormalities, and, rarely, the “diencephalic syndrome”, in which a disordered metabolism results in failure to thrive, severe emaciation, and near absence of subcutaneous fat.

## 4. Diagnostic Studies

Magnetic resonance (MR) imaging is the modality of choice for evaluating central nervous system neoplasms. Contrast-enhanced MR should be obtained at the time of tumor diagnosis. A number of low-grade gliomas do not demonstrate enhancement with gadolinium and contrast can be avoided in subsequent scans once this has been established, with the caveat that notable growth or other factors may provide a new indication for contrast administration to look for radiographic signs of malignant transformation. Computed tomography (CT) often first identifies an otherwise asymptomatic tumor when obtained for an unrelated reason, such as trauma. Unless there is some reason for which an MR cannot be obtained (such as a non-MR-compatible implant), any CT which is suspicious for a neoplasm should be followed by MR to better evaluate the anatomical borders and characteristics of the lesion.

Low-grade infiltrative astrocytomas are isodense or hypodense on CT scan, often without any enhancement which would be suggestive of a higher-grade tumor. They are iso- or hypointense on T1-weighted MR, hyperintense on T2-weighted MR, and suppress on FLAIR imaging. Again, contrast enhancement is generally not present. Pilocytic tumors classically are cystic with well-defined borders and an enhancing mural nodule, though a minority of tumors can be heterogeneously mixed solid and cystic or completely solid. Almost all demonstrate some degree of enhancement. Pleomorphic xanthoastrocytomas characteristically arise near the cortical surface, often with a cystic component and an enhancing mural nodule. They may exhibit a dural tail, leptomeningeal involvement, and scalloping of the overlying bone. The solid tumor component generally demonstrates intense enhancement. Subependymal giant cell astrocytomas frequently present with obstructive hydrocephalus and are seen as discrete, homogeneously enhancing lesions located near the foramen of Monro. Gangliogliomas may resemble other astrocytic tumors, but variably contrast-enhance on both CT and MR imagine and, more frequently, demonstrate calcification. Desmoplastic infantile gangliogliomas present as large superficial hemispheric masses which often invade the leptomeninges. These lesions often contain mixed cystic and vividly enhancing solid components which are isointense on T1- and T2-weighted MR imaging. Leptomeningeal involvement may be visible as a dural tail and calcification is variable.

Other diagnostic studies can provide information relevant to perioperative management. Neuraxis imaging is recommended for tumors which tend to seed the leptomeninges, or if suspicion for a genetic syndrome, such as NF1, has been raised. Similarly, lumbar puncture should be utilized if leptomeningeal spread is suspected or if imaging features lead to the suspicion of a radiosensitive germ line tumor or lymphoma. Formal diagnostic angiography is indicated if a tumor exhibits unusual vascularity. A comprehensive neuro-ophthalmologic evaluation is warranted for any tumors involving the visual pathway, including fundoscopy and evaluation of visual fixation in infants and an assessment of visual acuity and visual fields in older children. Hypothalamic, suprasellar, and pituitary tumors warrant a thorough endocrinologic evaluation, with replacement of any endocrine deficiencies. Stress dose corticosteroids during the perioperative period may be indicated in cases of relative hypocortisolemia. In older children, detailed neuropsychological assessments are indicated if there is concern for cognitive impairment or neurobehavioral issues that warrant ongoing management. If these additional studies would alter operative management, they should be completed preoperatively. If not, they can be completed postoperatively after histological diagnosis.

## 5. Surgical Treatment

### 5.1. Perioperative Management

The timing of surgery is determined by the radiographic characteristics of the lesion and the clinical condition of the child. The specific management approach varies somewhat between surgeons, therefore, we discuss our own institutional practice. Small lesions without mass effect may be treated on an elective basis. Large lesions without symptoms may be treated on the next available operating day. Tumors causing hydrocephalus require external ventricular drain placement; in many cases this can be accomplished at the time of the tumor resection. Children who present with severe symptoms from mass effect of the lesion should undergo urgent resection.

Whether or not the patient presents with a history of seizures, patients with intrinsic tumors are at risk for seizures in the perioperative period. An anticonvulsant, such as levetiracetam, is generally initiated at the time of diagnosis of any supratentorial intrinsic tumor [31]. Phenytoin is used at the time of surgery if the risk for intraoperative seizure is of concern and particularly if functional cortical mapping is employed during the operation. Anticonvulsants are continued for at least a week postoperatively and for several months in those patients who presented with seizures preoperatively. Patients with epilepsy who are well-controlled on a home regimen may be continued on that regimen during the perioperative period.

A corticosteroid such as dexamethasone is initiated preoperatively in cases of large tumors or any tumors with notable perilesional edema [32]. A loading dose of dexamethasone is given just prior to surgery and continued postoperatively with a taper ranging from three days to several weeks, depending on the amount of perilesional cortical edema anticipated after the resection. Preoperative topical antibacterial cleanses targeted at reducing the burden of skin and hair flora are initiated at least one day prior to surgery.

### 5.2. Surgical Planning

The primary goals of surgery are to obtain tissue to establish a histopathologic diagnosis and to remove as much tumor as possible while not introducing any new neurological deficits. Gross total resection should be the goal whenever possible. In general for low-grade gliomas, the gross total resection ten-year progression-free survival (PFS) exceeds 85%, where a subtotal resection results in less than 50% ten-year PFS [33]. Therefore, for pilocytic astrocytomas, even those in subcortical areas, superficial nonpilocytic astrocytomas, and benign neuroepithelial tumors, due to the major prognostic advantage with gross total or near total resection [30,33,34,35,36], particular care should be taken to remove the tumor as completely as possible. For poorly circumscribed, nonpilocytic, low-grade gliomas which infiltrate into the deep nuclei or other eloquent areas or cross the midline, gross total resection is not feasible. In these cases, an image-guided stereotactic biopsy or open debulking in the case of larger lesions with mass effect would be more appropriate.

Functional MRI (fMRI), diffusion tensor imaging, somatosensory evoked potentials (SSEPs), and extraoperative cortical functional mapping through subdural grid, strip, or stereotactic electroencephalography (sEEG) electrodes can identify speech, motor, vision, or other eloquent cortices preoperatively [37,38]. In cases where seizures are present preoperatively, electrocorticography (ECoG) also provides the opportunity to identify seizure foci, which can allow the surgeon to tailor not just an oncological resection, but to include any relevant epileptogenic cortex to optimize postoperative seizure control [39]. Some groups achieved acceptable control of seizures with straightforward lesionectomy [36,40], while others observed that patients with long-standing epilepsy exhibited improved seizure freedom without medication if peri- or intraoperative seizure focus mapping was used to guide an extended resection [39,41,42]. Preoperative electroencephalography (EEG), sEEG, or ECoG can localize epileptogenic foci and successful resection of the entirety of perilesional epileptogenic tissue can be confirmed with postresection ECoG. Intraoperative stereotactic neuronavigation, ultrasound, intraoperative functional mapping of eloquent cortices (with awake mapping generally employed only with children over ten years of age), and intraoperative MR imaging are modalities which can be variously employed to achieve the goal of maximal cytoreduction without introducing new neurological morbidity [39].

## 6. Adjuvant Therapy, Prognostic Factors, and Outcome

### 6.1. General Principles for Adjuvant Therapy for Low-Grade Gliomas

The prognosis for low-grade gliomas in children after gross total resection is so good that upfront adjuvant therapy is rendered undesirable (Table 2). The overall five-year survival rate for children with incompletely resected low-grade gliomas exceeds 90% [2,3,30,33,43]. The outcomes for adults are significantly worse, and this discrepancy likely reflects fundamental differences in the biology of the lesions in the two age groups. Whereas low-grade gliomas in adults tend to undergo malignant degeneration [2,3,30], some subtotally resected pediatric low-grade gliomas remain quiescent over long periods of time. The exact rate of malignant transformation of pediatric low-grade gliomas is difficult to characterize due to its low incidence in pediatric series. One study identified eleven patients with initial diagnoses of grade 1/2 glioma or grade 2 astrocytoma who underwent malignant transformation to either glioblastoma or other high-grade glioma at a median time of 5.1 years. None of the analyzed risk factors, including radiotherapy, were associated with malignant transformation. While the small number of pediatric patients undergoing malignant transformation precluded an accurate estimate of the rate of malignant progression, the long-term risk of malignant transformation in WHO grade 2 infiltrative astrocytoma was demonstrated to be less than 10%. The rate in older children appeared to be higher than for all patients, but no significant differences were identified given the small sample size [44].

From a molecular standpoint, it was observed that tumor cells with *BRAF* alterations may undergo senescence after an initial period of growth, suggesting that a subgroup of pediatric low-grade gliomas may exhibit decelerating growth kinetics over time [13]. Given these variable growth characteristics and the relative rarity of malignant progression, adjuvant therapy may be deferred and any residual tumor followed expectantly with serial imaging. Progression may be treated with repeat resection and subsequent adjuvant therapy if a gross total resection is not obtained [30,33,36,48]. A gross total resection is often unachievable for deep-seated, infiltrative tumors, which have worse prognoses than superficial lesions [33,45]. When a surgical cure is not possible, complex management of unresectable tumors relies on the use of targeted molecular agents, traditional chemotherapeutics, and radiation therapy. General principles for the management of radiotherapy (RT) and traditional chemotherapeutics are discussed here. Targeted molecular agents are specific to tumor type and are discussed in more detail in subsequent sections.

While radiotherapy is a mainstay for the treatment of malignant pediatric CNS tumors such as medulloblastoma and ependymoma, the overall benefit and optimal timing of treatment of residual pediatric low-grade gliomas with radiotherapy are not as clear. While radiotherapy confers a survival advantage for adults with subtotally resected low-grade gliomas [2,49], the same advantage is not seen in children undergoing total or near total tumor removal [50]. Since even children undergoing subtotal total tumor resection experience a high incidence of progression-free survival, radiotherapy is often reserved for unresectable or progressive disease. Furthermore, the morbidity associated with radiotherapy is heightened in the pediatric age group. Radiotherapy anecdotally seems to increase the incidence of malignant transformation in incompletely resected gliomas [30,51] and can result in cognitive delay, endocrinopathy, and vasculopathy [52,53,54].

In one series, radiotherapy after subtotal resection significantly impacted PFS with a median follow-up of over eight years. However, the nonirradiated group exhibited better overall survival (OS), because three of the patients who received irradiation developed malignant lesions within the treatment field, whereas none developed in patients who did not receive RT [30]. Further study is needed to optimize selection criteria for radiotherapy, however, as there does appear to be a role for risk stratification among candidates for RT. A recent study demonstrated that a high-risk subgroup of patients with either diffuse astrocytoma histology or midbrain/thalamic tumor location appeared to experience improved overall survival with early rather than delayed radiation therapy. This survival advantage occurred despite a secondary malignancy rate of 7% at 15 years, which was comparable with other reports of 6%–8% at 20–30 years [55]. The Children’s Oncology Group trial ACNS0221 evaluated pediatric low-grade glioma treated with conformal radiation therapy of 54 Gy in 30 fractions and a clinical target volume of 5 mm. Children younger than ten years who showed no response after at least one course of chemotherapy were included in the study. Of 85 eligible patients, five-year PFS was 71% ± 6% and OS was 93% ± 4%. Male sex and nonpilocytic astrocytoma histology and large tumor size were negatively associated with OS [56]. This dosing scheme results in an acceptable PFS without a high rate of marginal relapse and serves as a treatment option for surgically inaccessible tumors.

In addition to more nuanced patient selection, technical advances are changing the risk profile of RT. Conventional radiation may be utilized safely in children older than 10–12 years with large areas of unresectable disease [53]. For younger children, conformal RT using advanced treatment-planning techniques to spare normal structures may be used to reduce morbidity [57]. Proton therapy may have advantages over traditional photon therapy in both the short- and long-term, with potential for improved side effect profiles for neurocognitive, hearing, and neuroendocrine function, as well as secondary malignancies [58]. Stereotactic radiosurgery [59,60] and interstitial radiotherapy [61] may also play a role in the treatment of selected unresectable low-grade glioma in children. In a pilot study of stereotactic radiosurgery in children with progressive low-grade gliomas, progression-free survival rates of 82.5% at five years and 65% at eight years and an overall survival rate of 97.8% at five years and 82% at eight years were obtained [62]. 

The fact that the low-grade gliomas which are unresectable also are often large and arise in younger children poses challenges for treatment with radiation therapy. To avoid or delay the use of radiation in very young children, conventional chemotherapy has been utilized [45,63]. The Children’s Oncology Group (COG) A9952 study randomized two active regimens, carboplatin and vincristine versus 6-thioguanine, procarbazine, lomustine, and vincristine, for unresectable or progressive low-grade gliomas in children without NF-1. Patients with NF-1 received only carboplatin and vincristine due to concerns for alkylator-induced malignancies. Tumor progression was delayed in both regimens. Unfortunately, children without NF-1 generally experienced disease progression within five years of therapy [45]. A phase III randomized trial by the International Society of Pediatric Oncology (SIOP) consortium demonstrated that adding etoposide to carboplatin/vincristine did not improve survival [64]. Various other chemotherapy regimens were trialed, but none achieved sustainable long-term disease control [65].

### 6.2. Pilocytic Astrocytoma

Pilocytic astrocytomas (PAs) are one of the most frequently encountered brain tumors in the pediatric population, typically presenting between ages eight and thirteen [21]. PAs have a better prognosis than most other nonpilocytic gliomas. It is unclear whether this is due to the fact that pilocytic tumors are usually well-circumscribed and more amenable to gross total resection than nonpilocytic gliomas, or whether they represent a more biologically favorable tumor [2,30,33,36]. Regardless, pilocytic astrocytomas have excellent overall prognoses. After removal of all radiologically detectable tumor, the five-year PFS exceeds 75% and in many studies it approaches 100% [30,33,36,43,66]. Subtotally resected tumors which do not receive radiation therapy still have a five-year PFS of approximately 50% to 60% [30,33,43].

Many PAs exhibit translocations or activating mutations of the *BRAF* gene, or alterations in other components of the mitogen-activated protein kinase (MAPK) signaling pathway, such as *NF1* mutations and *RAF* fusions. *BRAF*–*KIAA* fusions, which lead to constitutive activation of the BRAF protein, are common in cerebellar and optic pathway pilocytic tumors, whereas *BRAF* mutations are more common in gangliogliomas, pleomorphic xanthoastrocytomas, and cerebral pilocytic astrocytomas [25]. Biologic agents which inhibit the MAPK signaling pathway are being developed as therapies for these tumors.

Selumetinib inhibits MEK1/2 (MAPK/ERK kinase, AstraZeneca, Cambridge, UK) and thereby MAPK activation. The Pediatric Brain Tumor Consortium (PBTC) conducted a phase I study of selumetinib and found that 5 of 25 low-grade gliomas had durable partial (>50%) responses to the agent and the majority had at least some tumor response [67]. A phase II study stratified patients by *BRAF* aberration status, histological diagnosis, tumor location, and NF-1 status. Based on the strong activity observed among WHO grade I pilocytic astrocytomas harboring either of the two most common BRAF aberrations (*KIAA1549-BRAF* fusion or *BRAF*^V600E^) and NF1-associated pediatric low-grade gliomas (WHO grades I and II), phase three studies comparing standard chemotherapy to selumetinib in patients with low-grade glioma with and without NF-1 were initiated [68]. Similarly, vemurafenib (NCT01748149, Genentech, San Francisco, CA, USA) and dabrafenib (NCT01677741, Novartis, Basel, Switzerland) target tumors with *BRAF^V600E^* mutations. A phase 2 randomized clinical trial (NCT02684058) compared the activity of dabrafenib and trametinib (a MEK inhibitor, Novartis, Basel, Switzerland) to the combination of carboplatin and vincristine in children with newly diagnosed *BRAF^V600E^*-mutated low-grade gliomas. Antiangiogenic agents such as bevacizumab and lenalidomide also have been trialed [22,23,69].

There is extensive literature incorporating cellular and animal models of these and other tumors which helped to elucidate the biochemistry of these signaling pathways, suggest therapeutic targets, and characterize the mechanisms of resistance for targeted drugs [70,71,72]. While an extensive discussion of these models is beyond the scope of this review, it is important to note the role that these studies may play in identifying future target drugs for human studies.

### 6.3. Diffuse Astrocytoma

Together with pilocytic astrocytomas, diffuse astrocytomas are among the most prevalent pediatric brain tumors [73]. While greater extent of resection and hemispheric tumor location are reliable predictors for good survival outcomes in all classes of low-grade astrocytomas, diffuse astrocytomas (DAs) have poorer outcomes than pilocytic astrocytomas. In one study, three-year PFS was 63% for PA and 40% for DA patients and five-year OS was 96% ± 2% for PA and only 48% ± 10% for DA patients [46]. While we are not aware of studies examining the prognostic implications of the subtypes of DA in children, adult patients with gemistocytic tumors experience less than half the median survival of those with fibrillary tumors (38 vs. 82 months) [74]. Adjuvant therapy for diffuse astrocytomas includes traditional chemotherapy and radiotherapy, as discussed above, as well as MAPK inhibitors for the subgroup of tumors with alterations in that signaling pathway.

### 6.4. Pleomorphic Xanthoastrocytoma

Like pilocytic astrocytomas, pleomorphic xanthoastrocytomas have an excellent long-term prognosis, with outcome largely determined by the extent of resection. Gross total resection results in 90% survival at five years and 80% at ten years versus 65% at five years for incompletely resected tumors [75,76]. Although they are usually low-grade lesions, approximately 20% progress to higher-grade tumors [77,78]. Necrosis and increased mitotic activity portend a worse outcome when seen [75,77,79]. Recurrent tumors sometimes demonstrate increased anaplastic features at reoperation, suggesting that a subset of these lesions harbor more potential for malignant progression. *BRAF*^V600E^ mutations and 9p21 (*CDKN2A/B*) deletions may be seen [9], which may make adjuvant treatment with *BRAF*-targeted therapies an option for residual or recurrent disease. 

### 6.5. Subependymal Giant Cell Astrocytoma

Subependymal giant cell astrocytomas arise in children with tuberous sclerosis. These lesions are located in or near the ventricles and often have an abundant vascular supply, which can result in significant morbidity when resected. Due to the risks inherent in surgical treatment of these lesions and their indolent nature, they require resection only if they attain a large size or obstruct the ventricular system. Total or near total resection results in an excellent prognosis [80]. Subtotally resected lesions tend to enlarge over time [81], however, recurrent tumors can be re-resected at a later date.

Unresectable recurrent lesions can be treated with radiotherapy or stereotactic radiosurgery, but the long-term efficacy of radiation therapy for these tumors remains to be defined. Molecular therapy targeting dysregulated mTOR signaling showed excellent short-term tumor control, with a reduction of at least 30% volume in 75% of patients and at least 50% in 32% of patients who received everolimus. Additionally, seizure frequency decreased and quality of life scores increased [22,82]. Another study of four pediatric patients with tuberous sclerosis treated with sirolimus saw decreased tumor size in all patients and improved seizure control in some patients at short-term follow-up [23]. The long-term efficacy of this therapy, particularly if used in lieu of surgery, requires further study.

### 6.6. Benign Neuroepithelial Tumors

Gangliogliomas are usually well circumscribed and amenable to complete resection, with a five-year survival rate exceeding 90% [33,83]. Similarly, dysembryoplastic neuroepithelial tumors are also well-circumscribed, indolent lesions amenable to complete resection with excellent long-term outcomes. Adjuvant therapy is utilized only for tumors which progress and are thought to be unresectable [84,85,86]. Gangliogliomas often harbor *BRAF* mutations, particularly *BRAF*^V600E^, or other alterations of the MAP kinase pathway [25,26]. A small subset exhibit *CDKN2A* deletion [26]. DNETs frequently possess *FGFR1* alterations and MAP kinase pathway activation is common [29]. Tumors which possess alterations in MAP kinase pathway activity may be candidates for molecularly targeted therapy. Both of these tumor types frequently present with medically intractable epilepsy [83,87], thus, efforts to include the resection of epileptogenic foci associated with the tumor should be undertaken to maximize seizure control and long-term functional outcomes [87].

Desmoplastic infantile gangliogliomas generally present with symptoms of increased intracranial pressure due to the rapid growth exhibited by this type of tumor. The large size and dense vascularity of these lesions, which are found in young infants without a large circulating blood volume, can preclude gross total resection. Whenever possible, complete resection provides the best chance for long-term PFS [88,89]. The treatment of any residual tumor is controversial. As with many other types of pediatric low-grade glioma, progression is not always the rule and spontaneous regression of the residual tumor may occur. Therefore, upfront adjuvant chemotherapy for patients with radiographically visible residual disease [90] or expectant management with repeat resection after demonstrated tumor progression [91,92] have both been recommended as appropriate treatment strategies. These tumors frequently exhibit *BRAF*^V600E^ mutations, which make targeted therapeutics an option for some cases of residual or recurrent tumor.

## 7. Conclusions

Overall, developments in surgical and imaging technology, conformal radiation therapy delivery, and conventional chemotherapy have advanced the treatment of low-grade gliomas, leading to improved outcomes. Surgery remains the treatment of choice for cerebral and cerebellar hemispheric tumors, with high rates of long-term survival after gross total resection. Unfortunately, complete surgical resection is often not possible for deep-seated tumors, such as optic gliomas. Historically, irradiation was used as a salvage treatment for progressive deep-seated tumors, but was associated with cognitive and endocrine morbidity since the tumors were often midline, large, and in young children. More recently, interest focused on using conventional chemotherapy as a way to delay or avoid the neurotoxic effects of irradiation. Technical advances in radiation oncology also reduced morbidity in necessary radiotherapy. During the last decade, the majority of childhood brain tumors were molecularly characterized and insights from these data inspired numerous molecular-targeted therapies, with it becoming apparent that many low-grade gliomas exhibit alterations in the MAPK signaling pathway, either in terms of *BRAF–KIAA1849* translocation, *BRAF*-activating mutations, NF1 gene deletions or mutations, and other less common genetic events that dysregulate this pathway. The availability of a host of agents to inhibit the MAPK pathway led to promising results in phase I and II trials and set the stage for recently developed phase III studies within the Children’s Oncology Group to compare molecular-targeted therapy with the best conventional chemotherapy regimens. Other molecular-targeted therapies are currently under study, providing hope that the future will see a host of new therapies to further improve the treatment of pediatric low-grade gliomas.

## Figures and Tables

**Table 1:** Summary of WHO classification of low-grade gliomas (adapted from [9]).

Tumor Class	Tumor Type	WHO Grade
Diffuse Astrocytic and Oligodendroglial Tumors	Diffuse astrocytoma	II
	Oligodendroglioma	II
Other Astrocytic Tumors	Pilocytic astrocytoma	I
	Subependymal giant cell astrocytoma	I
	Pleomorphic xanthoastrocytoma	II
Ependymal Tumors	Subependymoma	I
	Myxopapillary ependymoma	I
	Ependymoma	II
Other Gliomas	Angiocentric glioma	I
	Chordoid glioma of the third ventricle	II
Neuronal and Mixed Neuronal–Glial Tumors	Dysembryoplastic neuroepithelial tumor	I
	Gangliocytoma	I
	Ganglioglioma	I
	Dysplastic gangliocytoma of cerebellum (Lhermitte–Duclos)	I
	Desmoplastic infantile astrocytoma and ganglioglioma	I
	Papillary glioneuronal tumor	I
	Rosette-forming glioneuronal tumor	II

**Table 2:** Summary of histology, molecular features, management, and survival outcomes for select pediatric low-grade gliomas.

Tumor	Histology	Molecular Features	Management	Outcomes
Pilocytic astrocytoma	Compact bipolar astrocytes with long GFAP-positive processes, eosinophilic granular bodies, Rosenthal fibers, microcysts, leptomeningeal infiltration, glomeroid vascular proliferation, and mitoses.	A total of 80% exhibit *BRAF* gene or other MAPK signaling pathway alterations (*BRAF*^V600E^*, BRAF/KIAA1549* translocation, neurofibromin mutation, etc.).	Gross total resection (GTR) is the surgical goal. Biologic agents such as selumetinib, vemurafenib, dabrafenib and trametinib, as well as traditional chemotherapy and radiotherapy are treatment options for unresectable residual or recurrent disease.	After GTR, the five-year PFS is 75%–100%. Subtotally resected tumors have a five-year PFS of approximately 50%–80%.
Diffuse astrocytoma	Diffuse infiltration of well-differentiated neoplastic astrocytes. Mitotic activity is absent.	Amplification and/or rearrangement of *MYB/MYBL1* [18,19,20].	GTR can be curative. Subtotal or no resection may be treated with vincristine plus carboplatin or vinblastine monotherapy [45].	Five-year PFS of 55% and OS of 87%. Reviewed diagnosis shows three-year PFS of 40% and five-year OS of 48% [46].
Pleomorphic xanthoastrocytoma	Dense cellularity and nuclear atypia with pleomorphism and multinucleation, low mitotic index, lipid-rich “xanthomatous” astrocytes, extracellular reticulin, eosinophilic granular bodies, and lymphocytic infiltrate.	*BRAF*^V600E^ mutations and 9p21 (*CDKN2A/B*) deletions may be seen [9].	GTR is the goal. *BRAF*^V600E^ -targeted therapy such as vemurafenib/dabrafenib or other MAP kinase pathway-targeted therapy may be possible. Adjuvant therapy is utilized only for tumors which progress and are thought to be unresectable.	GTR results in 90% long-term survival at five years and 80% at ten years, versus 65% at five years for incompletely resected tumors.
Subependymal giant cell astrocytoma	Large gemistocytic, spindled, and ganglion-cell like astrocytes. Immunoreactivity for both glial and neuronal markers is often observed. Perivascular pseudopalisading may be seen, mitoses are not.	Dysregulation of mTOR signaling linked with tuberous sclerosis. Germline mutations in *TSC1* or *TSC2* in up to 20% of patients [47].	GTR is the goal. Molecular therapy targeting dysregulated mTOR signaling such as everolimus/sirolimus and radiotherapy or stereotactic radiosurgery are used for unresectable recurrence.	Total or near total resection results in an excellent prognosis. Subtotally resected lesions tend to enlarge over time.
Ganglioglioma	Highly differentiated binucleated ganglion cells in a background of astrocytes or oligodendrocytes.	*BRAF* alterations, particularly *BRAF*^V600E^, or downstream members of the MAP kinase pathway [25,26]. A small subset exhibit *CDKN2A* deletion [26].	GTR is the goal. *BRAF*^V600E^ -targeted therapy such as vemurafenib/dabrafenib or other MAP kinase pathway-targeted therapy may be possible. Adjuvant therapy is utilized only for tumors which progress and are thought to be unresectable.	Five-year survival rate exceeding 90%.
Dysembryoplastic neuroepithelial tumor	Nodules of oligodendroglial-like cells and/or focal cortical dysplasia intermixed with a looser textured component containing “floating neurons” in a mucinous matrix.	*FGFR1* alterations and MAP kinase pathway activation are frequent [29].	Surgical excision often curative. MAP kinase pathway-targeted therapy may be possible. Adjuvant therapy is utilized only for tumors which progress and are thought to be unresectable.	Favorable outcome, particularly after GTR. While seizure control after resection was studied, we are unaware of any large series evaluating survival outcome.
Desmoplastic infantile ganglioglioma	Dense, fibrous, desmoplastic stroma containing a mixture of neuroepithelial cells with both astrocytic and neuronal differentiation.	Frequently have *BRAF*^V600E^ mutations [28].	Surgical resection can be curative. The treatment of any residual tumor is controversial as spontaneous regression can occur.	Rare enough that no large series to evaluate outcome is known to the authors.
